# Healthcare Workers' SARS-CoV-2 Infections in Four Hospital Outbreaks during Delta Variant Prevalence in Sydney, Australia

**DOI:** 10.1155/2023/1806909

**Published:** 2023-09-14

**Authors:** Danielle Hutchinson, Mohana Kunasekaran, Haley Stone, Xin Chen, Ashley Quigley, Aye Moa, C. Raina MacIntyre

**Affiliations:** ^1^Biosecurity Program, The Kirby Institute, Faculty of Medicine, University of New South Wales, Sydney, NSW 2052, Australia; ^2^College of Public Service & Community Solutions, Arizona State University, Tempe, AZ 85004, USA

## Abstract

**Background:**

Healthcare workers (HCWs) are at risk of SARS-CoV-2 infections due to occupational exposure. The use of airborne personal protective equipment (PPE) significantly reduces this risk. In June 2021, an epidemic of the Delta variant began in New South Wales (NSW), Australia. Concurrent PPE guidelines, set by the Clinical Excellence Commission (CEC), restricted the use of respirators.

**Objective:**

To understand the relationship of PPE guidelines with workplace-acquired HCW SARS-CoV-2 infections in different clinical settings and to examine the relationship between rates of community transmission and workplace-acquired HCW infections during the Delta outbreak in NSW.

**Methods:**

Total SARS-CoV-2 HCW infections between 13 June and 30 October 2021 (first four months of the Delta wave) were estimated from the government COVID-19 surveillance reports and compared with the surveillance reports of community transmission. In the absence of a detailed reporting of HCW infections, open-source data including news articles, media releases, and epidemiological surveillance reports were also collected. Data were extracted on HCW cases of SARS-CoV-2 from four hospitals, including the number of HCW cases (per NSW Health definition), clinical setting, PPE guidelines, and evidence of increasing local transmission.

**Results:**

SARS-CoV-2 infections in HCW identified as workplace-acquired infections (*n* = 177) and those without a known transmission source (*n* = 532) increased during the period of increasing community transmission (*n* = 75,014) in NSW. Four hospital COVID-19 clusters affecting 20 HCWs were identified between June and October 2021. HCW clusters occurred in general wards where staff were recommended to wear surgical masks. No workplace-acquired HCW infections were reported in these hospitals from critical care wards, where respirators were recommended during the same outbreak weeks.

**Conclusions:**

Differences in PPE policy across different wards may leave healthcare staff at risk of SARS-CoV-2 infection. During periods of high community transmission, respirators should be provided to protect hospital staff. Formal reporting of HCW infections should occur.

## 1. Introduction

Since the emergence of COVID-19, the disease caused by the SARS-CoV-2 virus, in December 2019, more than 495 million cases have been confirmed globally [1]. In New South Wales (NSW), Australia, an epidemic of the Delta variant in Sydney, Australia, began in June 2021 and spread to all regions of NSW [2]. Healthcare workers (HCWs) are at higher risk of contracting COVID-19 due to occupational exposure and may have not have had access to adequate personal protective equipment (PPE) early in the pandemic due to the denial of airborne transmission of the virus [3].

The World Health Organization (WHO) has identified HCWs as a group at increased risk of becoming infected with SARS-CoV-2 due to the increased likelihood of exposure through contact with people infected with the virus [4]. This risk increases with increasing levels of community transmission [4]. In September 2021, WHO estimated HCW deaths due to COVID-19 between 80,000 and 180,000 globally, with a likely estimate of 115,500 deaths [4]. In the Australian context, it has been shown that HCWs have up to three times the risk of infection compared with the general population, and there have been multiple hospital outbreaks throughout the pandemic [5]. During the first six months of the pandemic, the rate of HCW infection was 90/100,000, the overall population infection was 34/100,000, and HCWs comprised of an estimated 6.03% of all reported infections [5]. In the Australian state of Victoria, over 4000 health and aged care workers became infected in 2020, with at least 69 percent of infections acquired at the workplace [6].

Transmission of SARS-CoV-2 is airborne [7]. In performing routine care with prolonged patient contact and working within a contaminated indoor environment, HCWs are at risk of COVID-19 exposure in the workplace [8]. HCWs may then cause ongoing transmission to their coworkers, patients, their families, and community members [8]. The use of masks and respirators, has been shown to significantly lower the rate of COVID-19 infection among HCWs [9].

The NSW Department of Health policy regarding the use of PPE is informed by the guidelines set by the Clinical Excellence Commission (CEC) [10]. During the COVID-19 pandemic, HCWs have been required to use “contact and airborne precautions” (P2/N95 respirator and eye protection) when providing care to patients with confirmed or suspected COVID-19, or if determined a close contact by the Public Health Unit (PHU) [10]. At the start of the Delta outbreak (version 1.1 of guidelines [10]), staff working in the critical care wards, including emergency departments, intensive care units, and designated COVID-19 wards, were required to use respiratory PPE, while staff working in other clinical areas (noncritical care) were required to wear surgical masks when performing patient care during periods of moderate community transmission [10]. These guidelines do not account for the presence of asymptomatic infection among staff and patients. Furthermore, restrictive guidelines and shortages of respirators in the NSW hospital system led to some frontline HCWs to use surgical masks or PPE that had not been fit-tested [11]. In NSW, there is no formal reporting of HCW infections. Therefore, in this study, media articles and other publicly available data were searched for reports of hospital outbreaks.

### 1.1. Objective

The objective of this study is twofold: first, to investigate the relationship between PPE guidelines and workplace-acquired HCW SARS-CoV-2 infections at different clinical settings within hospitals, in the absence of a formal reporting of HCW infections; and second, to examine the correlation between rates of community transmission and HCW infections.

## 2. Methods

### 2.1. Data Collection

In the absence of formal, public reporting of workplace-acquired HCW infections in NSW, open-source data can provide valuable information on workplace outbreaks, although it may be limited with regard to completeness of case information [5].

We searched for open-source reports of HCW clusters of SARS-CoV-2 infections in hospitals in NSW, Australia, between 13 June 2021 (the start of the epidemiological week in which the first case of Delta variant of SARS-CoV-2 was reported in NSW [12]) and 30 October 2021.

We conducted a study with a retrospective comparative analysis using open-source data to identify HCW clusters in the Delta variant epidemic in NSW in 2021. In addition, we compared the PPE policy implementation across the affected and nonaffected wards in the same hospitals.

#### 2.1.1. Identifying HCW Infections in NSW

**Figure 1:**
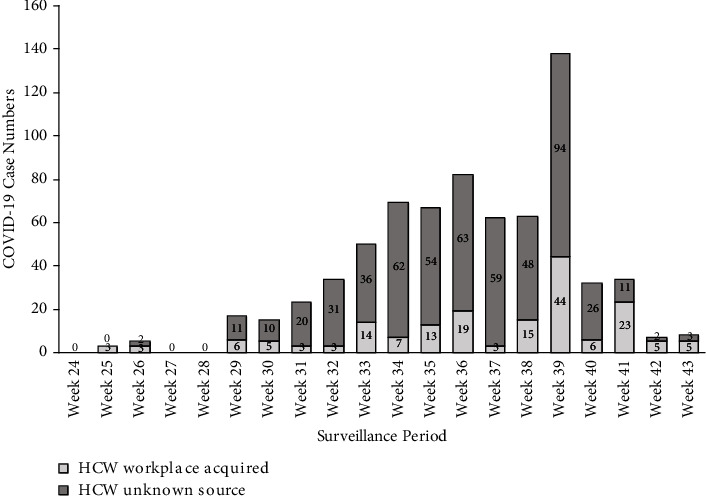
The number of HCW SARS-CoV-2 infections in NSW (13 June 2021 to 30 October 2021) (*n* = 890). Data source: COVID-19 in Healthcare workers in NSW. NSW Department of Health Health. COVID-19 weekly surveillance reports - Archive. NSW Department of Health. COVID-19 in NSW. NSW Department of Health. Coronavirus (COVID-19) case numbers and statistics. Australian Government Department of Health.

Total SARS-CoV-2 HCW infections between 13 June 2021 and 30 October 2021 were collated using information from the State COVID-19 surveillance reports (Table 1) [8, 12–14]. In NSW surveillance reports, HCWs were defined as medical and nursing staff, administrative and support staff, paramedics, laboratory technicians, pharmacists, and cleaners [2]. For this study, this definition is maintained. The state PHU designates the source of HCW infections following a case investigation [6]. Attribution to workplace acquisition of SARS-CoV-2 may be difficult in periods of high levels of community transmission; therefore, both “possible” and “unknown” cases (Table 1) have been included in Figure 1.

#### 2.1.2. Identifying HCW Workplace Outbreaks in NSW during the Delta Epidemic

Open-source data were collected for NSW, Australia, including media articles, hospital and state government media releases, and state government epidemiological surveillance reports, and were reviewed to identify workplace-acquired HCW infections between 13 June and 30 October 2021. The cohort study data collection began on 15 August 2021 and continued through 30 October 2021. A line list of reported positive cases was created with information including infection source, date of reported case, location including specific wards, policies relating to PPE, and vaccination coverage (see Table S1 in appendix). No identifying information was available or recorded. A Google search was conducted using the following keywords: (healthcare worker OR nurse OR hospital personnel) AND (NSW OR Sydney) AND (COVID-19 OR COVID OR coronavirus OR Delta OR SARS-CoV-2) AND (hospital OR ward) AND (outbreak OR cluster). The first six pages of each Google search were reviewed.

### 2.2. Inclusion and Exclusion Criteria for HCW Clusters

HCW clusters were defined as two or more cases of COVID-19 in the same healthcare facility within one week (Table 2). Critical care wards are defined as emergency departments, intensive care units, and designated COVID-19 wards, and noncritical care wards are defined as other wards providing clinical inpatient care.

HCW clusters were included if complete information was available about the hospital, the number of HCWs infected with documented transmission occurring at the workplace, dates, and the ward type (Table 2). The PPE policy was as per the CEC guidelines unless otherwise stated by the hospital. Vaccination coverage was obtained by open-source media reports or hospital press releases. To establish an increased likelihood of HCW exposure to COVID-19 across all wards (including emergency department presentations), a criterion for inclusion was that the cluster occurred in the week/s following a documented increase in community transmission (Table 2). This was determined by the designation of local government areas (LGAs) of concern or suburbs of concern by the NSW Department of Health, through manual search of the daily NSW Health media releases and the weekly epidemiological reports [12, 17]. NSW Health used COVID-19 case data, vaccination coverage, and movement trends in the local area to determine LGAs of concern [18]. Inclusion and exclusion criteria and the results are shown in Table 2.

### 2.3. Data Analysis

Analysis and reporting were performed by using Strengthening the Reporting of Observational Studies in Epidemiology (STROBE) guidelines for epidemiological studies [19]. Data collected for analysis focused on the HCW in four tertiary care hospitals located in Sydney during the Delta epidemic in 2021. The four hospitals were selected due to well-reported outbreaks among health workers, including publicly available details on the type of wards involved.

A descriptive analysis was conducted, and case counts were compared between noncritical care wards and critical care wards within the same hospital over the same period.

Total SARS-CoV-2 community and HCWs confirmed infections between 13 June 2021 and 30 October 2021 were collated using information from the state and commonwealth COVID-19 surveillance reports.

## 3. Results

### 3.1. HCW Infections in NSW

Total SARS-CoV-2 HCW infections between 13 June 2021 and 30 October 2021 are shown in Table 1 [8, 12–14]. There were 890 cases in HCW in that period. The source of HCW infections is designated by the state PHU following case investigation, without further information about how the source was ascertained [6]. The majority of HCW infections (*n* = 532) were classified as “unknown source,” followed by community-acquired cases (*n* = 181) and workplace-acquired cases (*n* = 177) [8, 12–14].

During epidemic periods, HCWs in NSW have had an increase in workplace-acquired infections (Figure 1). There had been 890 healthcare workers diagnosed with COVID-19 in the Delta outbreak by 30 October 2021 (epidemiological week 43), and of those, 42.1% (*n* = 375) were vaccinated with two doses of COVID-19 vaccine, 11.3% (*n* = 101) had received one dose, and 46.5% (*n* = 414) were unvaccinated [2]. The overall vaccination rate of HCWs in Sydney metropolitan hospitals as of 31 August 2021 was recorded as follows: 81% of HCWs had received two doses and 5% had received one dose [20].

### 3.2. HCW Workplace Outbreaks in NSW during Delta Epidemic

We identified six HCW outbreaks involving 25 infected HCWs, of which four met the inclusion criteria [21–27].  Table 3 shows that HCW outbreaks corresponded with the increasing community transmission by the local government area (LGA) [12, 28]. Vaccination rates were high compared to the community [20, 26, 29, 30]. The wards associated with the HCW clusters were general and noncritical care wards, including acute aged care, neurology, oncology, and orthopedic [21–27]. HCWs used surgical masks for patient care episodes on the affected wards, as per the CEC guidelines. Across the same weeks, there were no clusters identified in critical care wards (ICU and emergency), where HCWs were provided respirator masks for patient care [21–27].

#### 3.2.1. Hospital A 23–30 July 2021 (Epidemiological Week 31)

The hospital A cluster was reported on 24 July with the index case of a partially vaccinated nurse who worked across two wards whilst infectious, neurology and geriatrics [31]. During the week from 23 July to 30 July 2021 (epidemiological week 31), there were seven workplace-acquired infections among HCWs in these two wards [22, 23]. During the same period, there were no HCW infections in critical care wards [23], despite the likely exposure to COVID-19-positive patients due to the increasing community transmission. The relevant LGA was designated an LGA of concern from 18 July 2021 due to the rising community transmission [12]. Following this outbreak, all clinical staffs were required to wear full respiratory PPE [22]. At the time of the hospital A cluster, the vaccination rates for the staff were greater than 50% [29].

#### 3.2.2. Hospital B 13–20 August 2021 (Epidemiological Week 33)

The hospital B cluster was reported on 14 August, with the index case of an oncology patient who tested positive on 13 August [25]. Subsequently, five other oncology patients and two staff members were tested positive [25]. The staff members, a nursing unit manager and a junior medical officer, were both fully vaccinated [25]. During the same period, there were no reported HCW infections on critical care wards [20]. The surrounding area was designated an LGA of concern on 28 July 2021, due to the rising community transmission [32]. While vaccination rates were unable to be sourced for the relevant epidemiological week, it was documented on 31 August 2021 that across all Sydney metropolitan hospitals, staff vaccination rates were 81% fully vaccinated (two doses) and 5% partial vaccination (one dose) [20].

#### 3.2.3. Hospital C 14–21 August 2021 (Epidemiological Week 33)

The hospital C cluster was reported on 14 August, with an unknown index case. The first four cases of staff members in this cluster who tested positive for workplace-acquired COVID-19 were reported on 14 August [21]. During the week of 14–21 August (epidemiological week 33), eight HCWs were infected across the oncology and orthopedic wards; however, no HCW infections were reported in the critical care wards in the hospital [15, 21, 26]. The surrounding area was designated an LGA of concern from 8 August [33]. Following the outbreak, the hospital C required all staff to use respiratory protection during clinical interactions [21, 27]. Staff vaccination rates at hospital C were reported to be 70% at the time [26].

#### 3.2.4. Hospital D 9 September 2021 (Epidemiological Week 36)

The hospital D cluster was reported on 9 September, when two patients and three nurses tested positive in the geriatric ward [24]. Following this outbreak, staff were required to increase PPE levels for safety purposes [24], and no further cases were reported. During the same period of increasing community transmission in the surrounding area [28], there were no workplace-acquired infections reported in the critical care wards of hospital D. Staff vaccination coverage was 92% at the relevant time [30].

## 4. Discussion

There is variable public reporting of HCW infections due to workplace exposure to SARS-CoV-2. Due to this, we utilized open-source data to extract more detailed information on workplace outbreaks [5]. There were 171 HCW infections attributed to workplace-acquired sources by NSW Health, and this study examines outbreaks involving 20 of those cases. This is a descriptive study; therefore, while associations may be drawn, positive correlations with statistical significance have not been established.

We have shown that differences in PPE policy across different wards may contribute to healthcare staff risk of SARS-CoV-2 infection. The guidelines in operation during the study period recommended airborne precautions in critical care wards and no workplace-acquired infections in these settings were reported in open-source media or publicly available reports. In contrast, reported outbreaks occurred in general wards (geriatric, neurology, oncology, and orthopedics) where surgical masks were recommended during the same periods of high community transmission. Exposure of HCWs in emergency departments, intensive care units, and COVID-19 wards was likely, due to the high levels of community transmission; however, there were no HCW clusters reported in open-source media or publicly available reports in these clinical areas during that time. The guidelines used in NSW assumed that airborne transmission only occurs in selected situations such as aerosol-generating procedures; however, SARS-CoV-2 is airborne [7]. Approximately 35% of infections present as asymptomatic [34], and in addition, the presymptomatic stage of infection is highly contagious [35]; therefore, in times of increasing community transmission, precautionary PPE should be available. With a high rate of hospital staff vaccination in the Sydney metropolitan area, it is possible that the differences seen in this study could be attributed to differences in the types of PPE used in the differing clinical areas.

Australian HCWs are up to three times more likely to have SARS-CoV-2 infection than the general population [5, 6]. The Victorian second wave in 2020 reported that 63.4% of cases were acquired in a healthcare setting, 19.3% in the community, and 17.3% were unable to be determined [6]. In NSW, there was less information about attribution and almost half were classified as “unknown” sources. This suggests a potential attribution bias, which may function to minimize the true picture of workplace transmission in NSW hospital settings, and could underestimate the impact of inadequate use of PPE.

There were high rates of HCW vaccination in NSW, in part due to eligibility, and also as a public health order mandating COVID-19 vaccination at least one dose of a COVID-19 vaccine by 30 September 2021 and both doses by 30 November 2021, as a condition of employment [36]. At the time, two doses of the COVID-19 vaccine were considered “fully vaccinated.” By 31 August 2021, staff in metropolitan hospitals in Sydney had a vaccination rate of 81% vaccinated with two doses and 5% partial vaccination (one dose) [20]. However, it has been shown in other locations that even in highly vaccinated healthcare workforce, vaccine effectiveness decreases over time, in part due to characteristics of the circulating variant and in part due to waning immunity [37]. Vaccination should not be the only focus of COVID-19 control in high-risk settings such as hospitals, and respirators should be prioritized to protect HCW [37].

The WHO formally acknowledged that SARS-CoV-2 was spread through airborne transmission in May 2021 [7]. During times of increasing community transmission, HCWs are at a higher risk of acquiring workplace infections [5]. Changes in guidelines governing the provision of PPE can be expected to follow. In June 2021, the Infection Control Expert Group (ICEG) published national guidelines for the use of face masks by HCW in Australia [38]. The guidelines stated that respirator and eye protection should be used if the patient was known or suspected to be COVID-19 positive, if there was a current transmission in the community (especially if there were unlinked cases), if the duration of care was prolonged or at close proximity if there were other transmission risk factors such as the patient coughing or shouting, or if there was inadequate ventilation or sudden air movements (including a door opening or closing) [38].

The Clinical Excellence Commission in NSW updated the infection control and PPE guidelines on several occasions in 2021; however, the recommendations regarding the use of N95/P2 respirators did not change [10]. Therefore, it remained in the NSW hospital system that airborne protection (the use of N95 and eye protection) was to be used with patients with suspected or confirmed COVID-19 or close contact with COVID-19 cases as determined by the PHU [10].

These guidelines may not have been sufficient to protect HCWs in noncritical care wards during the pandemic [11]. Following the HCW clusters and severe staffing shortages, the relevant local health districts (LHDs) moved independently to require their workforce to wear PPE during all clinical interactions, overriding the CEC guidelines [21, 22, 24, 27]. Burnout, illness, and mass furloughing have created severe health workforce shortages during the pandemic. Therefore, there is a benefit beyond the protection of the individual to mitigating the airborne transmission of SARS-CoV-2 in healthcare facilities.

## 5. Limitations

The lack of formal reporting on health worker infections necessitated the use of open-source data for this study. Although the number of HCW cases was provided in Health bulletins, other information was not available, and the attribution of the source of infection was not described, with the majority of cases classed as “unknown.” Compliance with the outbreak measures by HCWs was also assumed and it is unknown what strategies were employed to measure it. The quantitative measure of compliance rates and qualitative data on the reasons for compliance with infection control measures could provide more depth to our understanding of the transmission of the SARS-CoV-2 virus among HCWs in a tertiary hospital setting. Further limitations to the study include the use of open-source data, including media articles, government reports, and documents, which may vary in completeness of case information and impact validity. The quality of the data used may be further improved by verification of the open-source data by hospital authorities or formal reports being issued, similar to the state of Victoria. This study conducted a descriptive analysis by comparing case counts of workplace-acquired HCW SARS-CoV-2 infections in noncritical care wards and critical care wards within the same hospital over the same period, as well as investigating HCW infections at times of increasing community transmission. While the variables under study (lack of policy requirement to use respirators in the work environment and workplace-acquired infection and community transmission and HCW infections) appear to have a relationship, no statistical analysis was performed, and therefore, a positive correlation between the variables cannot be concluded.

## 6. Conclusions

In this descriptive study, we analysed four hospital outbreaks affecting 20 HCWs during the Delta wave of SARS-CoV-2 in Sydney, Australia. All the identified HCW clusters were in general wards who utilized surgical masks as PPE. Differences in PPE policy across different wards may leave healthcare staff at a disproportionate risk of SARS-CoV-2 infection. All hospital HCWs should be provided with respirators during periods of increasing community transmission of SARS-CoV-2. To meet work health and safety obligations toward HCW, healthcare-acquired infections should be reported.

## Figures and Tables

**Figure 2:**
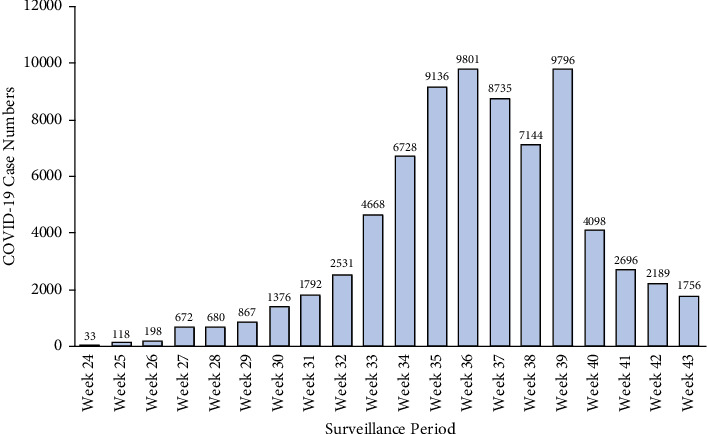
The total number of SARS-CoV-2 infections in NSW (13 June 2021 to 30 October 2021) (*n* = 75,014). Data source: COVID-19 Weekly Surveillance in NSW, NSW Department of Health, 2021.

**Table 1:** NSW HCW SARS-CoV-2 infections by attributed source (13 June 2021 to 30 Oct, 2021) [8, 12–14].

Reporting period	13 June 2021 to 30 October, 2021 (epidemiological weeks 25–43)
Dominant strain	Delta
Vaccination	High rates of vaccination among HCW
Workplace-acquired cases^*∗*^	177
Community-acquired cases^*∗*^	181
Unknown source^*∗*^	532
Total HCW with COVID-19	890
Total cases with COVID-19 in NSW	75,014

^*∗*^As assessed by NSW Health. Criteria for attributing place of infection not available.

**Table 2:** Inclusion and exclusion criteria and results.

Inclusion criteria	Included HCW clusters
Two or more HCWs in the same cluster were infected within a one-week period. Complete information about the hospital, the number of HCW infected with documented transmission occurring at the workplace, dates, and the ward type, in the week/s following an increase in community transmission, determined by the designation of LGA of concern	Hospital A (*n* = 7) Hospital B (*n* = 2) Hospital C (*n* = 8) Hospital D (*n* = 3)

*Exclusion criteria*	*Excluded*

No information about workplace transmission, less than 2 HCW infected, and no information about ward type	Hospital E: 24 patients and five staff members were infected across six separate wards (*n* = 5) in an epidemiological week. No information about workplace transmission [15] Hospital F: one patient and one HCW (*n* = 1) [16]

**Table 3:** Summary of HCW clusters in NSW during Delta epidemic 2021 (epidemiological weeks 24–43).

HCW cluster	Hospital A	Hospital B	Hospital C	Hospital D
Local area identified as increasing community transmission^*∗*^	18 July 2021	28 July 2021	8 August 2021	7 September 2021
Outbreak date	23–30 July 2021 *epidemiological week 31*	13–20 August 2021 *epidemiological week 33*	14–21 August 2021 *epidemiological week 33*	9 September 2021 *epidemiological week 36*
Vaccination rate	>50%	Approx. 80%	70%	92%
Wards associated with cluster	Acute aged care, neurology (29 patients)	Oncology (6 inpatients)	Oncology, orthopedic	Acute aged care

*Number of HCW with workplace-acquired SARS-CoV-2 infection*				
Noncritical care wards	7	2	8	3
Critical care wards	0	0	0	0

*PPE policy in place at the commencement of the outbreak*				
Noncritical care wards	Surgical masks for patient care (as per CEC guidelines)	Surgical masks for patient care (as per CEC guidelines)	Surgical masks for patient care (as per CEC guidelines)	Surgical masks for patient care (as per CEC guidelines)
Critical care wards (ED, ICU, and COVID-19 wards)	Contact and airborne precautions (respirator mask)	Contact and airborne precautions (respirator mask)	Contact and airborne precautions (respirator mask)	Contact and airborne precautions (respirator mask)

^*∗*^Determined by NSW department of health.

## Data Availability

The data that support the findings of this study are derived from public resources, namely, media articles, press releases, and government surveillance reports, which are openly available at locations cited in the reference section.

## Supplementary Materials

Supplementary Materials Table S1 contains details of reported positive cases including infection source, date of reported case, location including specific wards, policies relating to PPE and vaccination coverage, and the data source with corresponding uniform resource locator (URL).
