# Supplementary material for: Healthcare Workers' SARS-CoV-2 Infections in Four Hospital Outbreaks during Delta Variant Prevalence in Sydney, Australia
Source: Nurs Res Pract. 2023 Sep 14;2023:1806909. doi: 10.1155/2023/1806909 (PMC10513866; doi:10.1155/2023/1806909)
Supplement: Supplementary Materials — Table S1 contains details of reported positive cases including infection source, date of reported case, location including specific wards, policies relating to PPE and vaccination coverage, and the data source with corresponding uniform resource locator (URL). [file 1806909.f1.docx]

**Table S1: Data sources**

| Hospital Outbreak (de-identified) | Epi week | Source | Type | URL | Date updated | Index case if known | No. Infected HCW | Setting | PPE protocol for affected clinical area | No. infected critical care | Community transmission | HCW vaccination coverage | Other |
| --- | --- | --- | --- | --- | --- | --- | --- | --- | --- | --- | --- | --- | --- |
| A | 31 | Ward M. Tragedy as three more die after catching COVID-19 at Liverpool Hospital. ABC News. | News media | <https://www.smh.com.au/national/nsw/tragedy-as-three-more-die-after-catching-virus-at-liverpool-hospital-20210807-p58gpu.html> | 7-Aug | Partially vaccinated nurse |  | Neurology and geriatrics |  |  |  |  |  |
| A | 31 | Jansen J. Liverpool Hospital Covid cluster: Doctor’s plea for others to get vaccinated. The Daily Telegraph. | News media | <https://www.dailytelegraph.com.au/coronavirus/liverpool-hospital-covid-cluster-doctors-plea-for-others-to-get-vaccinated/news-story/5f8fdc900cccfdfb92a9e52fd2ae7205> | 16-Aug |  | 7 in total |  |  |  |  |  | in-hospital PPE protocol - all clinical staff to wear respirators following outbreak |
| A | 31 | Davies A. NSW records 172 new Covid cases as Berejiklian flags greater Sydney lockdown lasting into September. The Guardian. | News media | <https://amp.theguardian.com/world/2021/jul/27/nsw-covid-update-172-new-cases-as-berejiklian-flags-greater-sydney-lockdown-lasting-into-september> | 27-Jul |  | 3 |  |  |  |  |  |  |
| A | 31 | COVID-19 weekly surveillance reports - Archive. NSW Department of Health. | State government report | <https://www.health.nsw.gov.au/Infectious/covid-19/Pages/weekly-reports-archive.aspx> | Aug-21 |  |  |  |  | 0 | LGA of concern 18 July 2021 |  |  |
| A | 31 | District News. South Western Sydney Local Health District. | hospital media release | <https://www.swslhd.health.nsw.gov.au/pdfs/newsletters/2021Jul.pdf> | Jul-21 |  |  |  |  |  |  | 50% |  |
| A | 31 | COVID-19 Infection Prevention and Control Manual for acute and non-acute healthcare settings. Sydney Australia: Clinical Excellence Commission. NSW Clinical Excellence Commission. | State government Department of Health policy manual | <https://www.cec.health.nsw.gov.au/keep-patients-safe/COVID-19/COVID-19-IPAC-manual> | Jul-21 |  |  |  | surgical mask |  |  |  |  |
|  |  |  |  |  |  |  |  |  |  |  |  |  |  |
| B | 33 | Porter M. No new cases linked to St George Hospital oncology ward COVID-19 cluster. The Leader. | News media | <https://www.theleader.com.au/story/7407999/no-new-cases-linked-to-hospital-cluster/> | 30-Aug-21 | oncology patient, tested positive 13 August | 2 | Oncology ward |  |  |  |  |  |
| B | 33 | Carroll L, Ward M, Cormack L. One in five health workers yet to receive first dose as deadline nears. The Sydney Morning Herald. | News media | <https://www.smh.com.au/national/nsw/one-in-five-health-workers-yet-to-receive-first-dose-as-deadline-nears-20210831-p58nj0.html> | 31-Aug-21 |  |  |  |  | 0 |  | 81% |  |
| B | 33 | Certainty for the community as restrictions adjusted and vaccines ramped up. NSW Government Department of Health. | State government media release | <https://www.nsw.gov.au/media-releases/certainty-for-community-as-restrictions-adjusted-and-vaccines-ramped-up> | 28-Jul-21 |  |  |  |  |  | LGA of concern 28 July 2021 |  |  |
| B | 31 | COVID-19 Infection Prevention and Control Manual for acute and non-acute healthcare settings. Sydney Australia: Clinical Excellence Commission. NSW Clinical Excellence Commission. | State government Department of Health policy manual | <https://www.cec.health.nsw.gov.au/keep-patients-safe/COVID-19/COVID-19-IPAC-manual> | Jul-21 |  |  |  | surgical mask |  |  |  |  |
|  |  |  |  |  |  |  |  |  |  |  |  |  |  |
| C | 33 | Ward M. Patients catch COVID-19 in Sydney oncology ward, Nepean cases triple. The Sydney Morning Herald | news media | <https://www.smh.com.au/national/nsw/patients-catch-covid-19-in-sydney-oncology-ward-nepean-cases-triple-20210816-p58j6w.html> | 16-Aug-21 | unknown | 4 | oncology |  | 0 |  |  |  |
| C | 33 | Dodds T. COVID latest: What’s going on at Nepean Hospital? | news media | <https://westernweekender.com.au/2021/08/covid-latest-whats-going-on-at-nepean-hospital/> | 16-Aug-21 |  | 4 | Oncology  (11 patients in total) |  | 0 |  | 70% |  |
| C | 33 | COVID-19 Weekly Surveillance in NSW Epidemiological Week 42, Ending 25 October 2021. NSW Department of Health. | State government surveillance report | <https://www.health.nsw.gov.au/Infectious/covid-19/Documents/covid-19-surveillance-report-20211025.pdf> | 25-Oct-21 |  |  |  |  |  | LGA of concern 8 August 2021 |  |  |
| C | 33 | Coronavirus (COVID-19) Nepean Hospital COVID-19 Update 30 August 2021. NSW Department of Health. | Hospitalmedia release | <https://www.nbmlhd.health.nsw.gov.au/nbmlhd-news/from-the-expert/nh-covid-19-update> | 30-Aug-21 |  | 8 in total | oncology and orthopedic |  | 0 |  |  |  |
| C | 31 | COVID-19 Infection Prevention and Control Manual for acute and non-acute healthcare settings. Sydney Australia: Clinical Excellence Commission. NSW Clinical Excellence Commission. | State government Department of Health policy manual | <https://www.cec.health.nsw.gov.au/keep-patients-safe/COVID-19/COVID-19-IPAC-manual> | Jul-21 |  |  |  | surgical mask |  |  |  |  |
|  |  |  |  |  |  |  |  |  |  |  |  |  |  |
| D | 36 | COVID-19 cases at St Vincent's Hospital. abc News | News media | <https://www.abc.net.au/news/2021-09-10/sydney-news-staff-patients-contract-covid-19-st-vincent-hospital/100448124> | 10-Sep-21 | not stated | 3 | Geriatric  (2 patients) |  | 0 |  |  | all staff required to increase PPE for safety purposes |
| D | 36 | Thorpe N. Inside new COVID-19 battlegrounds of Sydney's inner-city suburbs of Redfern, Glebe, Waterloo. abc News. | News media | <https://www.abc.net.au/news/2021-09-15/covid-19-growing-inside-redfern-waterloo-glebe/100461558> | 15-Sep-21 |  |  |  |  |  | increased community transmission in surrounding area |  |  |
| D | 36 | Carroll L, Ward M. Unvaccinated nurses bring heart procedures to standstill in Sydney hospital. The Sydney Morning Herald. | News media | <https://www.smh.com.au/national/nsw/unvaccinated-nurses-bring-heart-procedures-to-standstill-in-sydney-hospital-20210916-p58s7v.html> | 17-Sep-21 |  |  |  |  |  |  | 92% |  |
| D | 31 | COVID-19 Infection Prevention and Control Manual for acute and non-acute healthcare settings. Sydney Australia: Clinical Excellence Commission. NSW Clinical Excellence Commission. | State government Department of Health policy manual | <https://www.cec.health.nsw.gov.au/keep-patients-safe/COVID-19/COVID-19-IPAC-manual> | Jul-21 |  |  |  | surgical mask |  |  |  |  |
